# Sea squirt alternative oxidase bypasses fatal mitochondrial heart disease

**DOI:** 10.15252/emmm.201809962

**Published:** 2018-12-10

**Authors:** Ann Saada

**Affiliations:** ^1^ The Monique and Jacques Roboh Department of Genetic Research Department of Genetics and Metabolic Diseases Hadassah Medical Center Jerusalem Israel; ^2^ Faculty of Medicine Hebrew University of Jerusalem Jerusalem Israel

**Keywords:** Cardiovascular System, Genetics, Gene Therapy & Genetic Disease

## Abstract

Mitochondrial diseases are a diverse group of inborn disorders affecting cellular energy production by oxidative phosphorylation (OXPHOS) via the five (CI‐CV) mitochondrial respiratory chain (MRC) complexes. The sea squirt alternative oxidase (AOX) is able to bypass the distal part of the MRC and was shown to alleviate the consequences of CIII and CIV defects in several cellular and *Drosophila* models. In this issue of *EMBO Molecular Medicine*, Rajendran *et al* (2019) demonstrate the first proof of concept in mammals, by showing that AOX is capable to extend lifespan and prevent heart failure in a CIII deficient mouse model, raising the possibility of future human AOX bypass treatment.

Mitochondria are intracellular organelles with a separate genome (mtDNA) and translation system, present in all enucleated cells. They are involved in numerous cellular pathways (intermediate metabolism, iron and calcium metabolism, cell death, etc.), but their main function is to provide cellular energy (ATP) via the mitochondrial respiratory chain (MRC), which is composed of ~89 proteins in five multimeric complexes (CI‐CV, with CI, CIII, CIV as super complexes) embedded in the mitochondrial inner membrane (IM). CI and CII transfer electrons from NADH and FADH_2_ originate from the tricarboxylic acid (TCA) to coenzyme Q (Q, ubiquinone). Q also subsequently receives electrons from additional pathways (pyrimidine synthesis, fatty acid oxidation and glycolysis); electrons from reduced Q (QH_2,_ ubiquinol) are transferred via CIII, cytochrome *c* and CIV (cytochrome *c* oxidase) to the final electron acceptor oxygen, forming water. Simultaneously, protons are translocated across the IM by CI, CIII and CIV, creating an electrochemical gradient (proton‐motive force), which is utilized by CV (ATP synthase) to generate ATP. This oxygen‐requiring process, termed oxidative phosphorylation (OXPHOS), provides the vast majority of the cell's energy requirements. Under normal conditions, a small fraction of the electrons escape the MRC and form oxygen free radicals (ROS), which may participate in cell signalling (El‐Khoury *et al*, 2016; Fig 1A).

**Figure 1:**
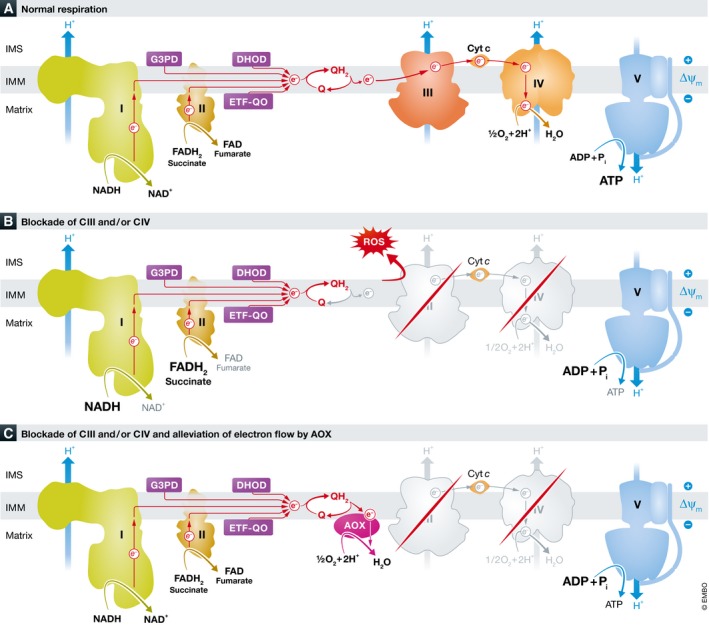
Simplified scheme of the mitochondrial respiratory chain and bypass CIII and CIV by AOX (A) Normal respiration, (B) blockade of CIII and/or CIV, (C) alleviation of electron flow by AOX. G3PG: glycerol‐3‐phosphate dehydrogenase, DHD: dihydroorotate dehydrogenase, ETF‐QO: electron transfer flavoprotein dehydrogenase, Cyt *c*: cytochrome C, Q: coenzyme Q ROS: oxygen free radicals, AOX: alternative oxidase, IM: mitochondrial inner membrane.

Mitochondrial diseases affecting the OXPHOS are a heterogeneous group of prevalent genetic disorders transmitted either by maternal inheritance due to mutations in mtDNA genes (encoding 13 MRC core subunits, 2 mRNAs, 22 tRNAs) or by Mendelian inheritance due to mutations in nuclear genes encoding the remaining MRC subunits or one of the numerous assembly, transcription, translation and replication factors needed for mtDNA and OXPHOS maintenance. The clinical manifestations are extremely variable, can occur at any age and involve several organs, mainly highly energy‐dependent, such as brain, heart, muscle, liver and kidneys. OXPHOS dysfunction is also implicated in common neurodegenerative disorders including Parkinson's disease (PD) and Alzheimer's disease (AD). The cellular consequences of impaired OXPHOS are complex and include energy‐deficit oxidative stress by overproduction of ROS, accumulation of toxic metabolites and metabolic derangement (Suomalainen & Battersby, 2018; Fig 1B). Therapy is challenging and is mainly supportive by administration of Q and vitamins (mostly MRC co‐factors), although numerous pharmacological and genetic options are currently under intensive investigation (Garone & Viscomi, 2018).

An original idea for mitochondrial disease therapy, elaborated by Pierre Rustin and Howard Jacobs, was to bypass blockade in the distal (CIII‐CIV) part of the electron transport by alternative oxidase (AOX) (Fig 1C). This peculiar enzyme, found in plants and several lower animals, is capable of transferring electrons from QH_2_ directly to oxygen without proton translocation and is unaffected by cyanide, in contrast to the cyanide‐sensitive cytochrome c oxidase (CIV). Indeed, allotropic expression of the single peptide AOX from a sea squirt, *Ciona intestinalis,* conferred cyanide resistance and restored electron flow to CIV‐deficient cells in culture (Hakkaart *et al*, 2006; Dassa *et al*, 2009). AOX expression was also able to complement and alleviate several fruit fly *Drosophila* models of COX deficiency, and interestingly as well as models of PD and AD. Although some flies with complete loss of CIV activity or mutated mito‐ribosomal protein were not rescued, the results still implicated a wide‐spectrum therapeutic use of AOX (Fernandez‐Ayala *et al*, 2009; Kemppainen *et al*, 2014; El‐Khoury *et al*, 2016). Moreover, AOX expression was well tolerated in mice, with only minor phenotypic effects on the OXPHOS, but without any apparent negative effect on physiology (Szibor *et al*, 2017).

In this issue of *EMBO Molecular Medicine*, Rajendran and colleagues provide the first proof of concept that AOX bypass alleviates the pathological manifestations and expands lifespan in a relevant genetic mouse model of a human mitochondrial disorder (Rajendran *et al*, 2019). The researchers crossed AOX into a mouse model of CIII deficiency, which recapitulates many of the manifestations related to human GRACILE (foetal growth, restriction, aminoaciduria, cholestasis, liver iron overload, lactic acidosis and early death during infancy) syndrome caused by mutations in *BCS1L*, encoding a CIII assembly factor (Fernandez‐Vizarra & Zeviani, 2015). Lifespan of the CIII‐deficient AOX mice was markedly extended from ~7 to ~19 months, and lethal cardiomyopathy was prevented. Even though the effect appeared to be tissue‐specific, as liver disease was still present, kidney and brain manifestations were ameliorated and growth was restored. Heart mitochondrial ultrastructure, respiration and normalized metabolic alterations confirmed that AOX is indeed capable to bypass the OXPHOS defect.

Although restoration of the specific mutation by viral transfer or gene editing would theoretically be a more precise approach, AOX could provide a universal treatment strategy for all mutations affecting CIII and CIV, and possibly other diseases involving OXPHOS. The precise mechanism of AOX action has not yet been fully elucidated, but could include reducing oxidative and cellular stresses, and preventing NAD^+^ deficit by relieving the accumulation of stalled electrons, albeit at the expense of less efficient energy production. An additional advantage of AOX is that the enzyme becomes active only when needed, i.e., when a significant amount of QH_2_ accumulates, enabling fine‐tuning according to specific tissue demands. Whether AOX or other alternative enzyme bypass therapies are feasible in humans is presently “unchartered territory”, but will most probably be explored in the near future.

## Conflict of interest

The author declares that he has no conflict of interest.
